# Prevalence and Risk Factors for Echinococcal Infection in a Rural Area of Northern Chile: A Household-Based Cross-Sectional Study

**DOI:** 10.1371/journal.pntd.0003090

**Published:** 2014-08-28

**Authors:** Gerardo Acosta-Jamett, Thomas Weitzel, Belgees Boufana, Claudia Adones, Andrea Bahamonde, Katia Abarca, Philip S. Craig, Ingrid Reiter-Owona

**Affiliations:** 1 Instituto de Medicina Preventiva Veterinaria, Facultad de Ciencias Veterinarias, Universidad Austral, Valdivia, Chile; 2 Laboratorio Clínico, Clinica Alemana de Santiago, Facultad de Medicina Clinica Alemana, Universidad del Desarrollo, Santiago, Chile; 3 Cestode Zoonoses Research Group, School of Environment and Life Sciences, University of Salford, Salford, United Kingdom; 4 Unidad de Zoonosis, Secretaría Regional Ministerial de Salud, Región de Coquimbo, Coquimbo, Chile; 5 Infectious Diseases and Molecular Virology Laboratory, Pontificia Universidad Católica de Chile, Santiago, Chile; 6 Institute of Medical Microbiology, Immunology and Parasitology, University Clinic Bonn, Bonn, Germany; Ege University, Turkey

## Abstract

**Background:**

Hydatidosis is a zoonotic disease of worldwide distribution caused by *Echinococcus granulosus*. Our study aimed to determine the prevalence of human and canine echinococcosis as well as the associated risk factors in a rural area of the Limarí province in northern Chile.

**Methodology/Principal Findings:**

A cross-sectional study was conducted between August and November 2009 using a stratified sampling design in each of the five districts of the province. In the selected villages, up to 10 households were sampled. Serum and fecal samples from an adult family member and a dog were collected from each participating household. Risk factors were assessed by standardized questionnaires. Seroprevalence was assessed using a multi-step approach: an ELISA for screening, IFA, IHA and western blot for confirmation of results, respectively. The prevalence of echinococcal infection in dogs was determined by coproantigen genus specific ELISA. Chi-square, Fisher tests and logistic regressions were used to assess risk factors for human seropositivity and dog copropositivity. A seroprevalence of 2.6% (10/403) and coproprevalence of 28% (26/93) was recorded for humans and dogs respectively. Contact with dogs and dog feces were risk factors for human seropositivity while dog copropositivity was associated with home slaughter of livestock (OR = 3.35; CI 90%: 1.16–6.85) and households de-worming dogs (OR = 2.82; CI 90%: 1.33–8.43).

**Conclusions/Significance:**

Echinococcal infection of humans and their dogs is common in Limarí province. Risk factors for human seropositivity were related to contact with domestic dogs and their feces, whereas those for dogs were home slaughter of livestock and the practice of de-worming dogs.

## Author Summary

Hydatidosis is a hyperendemic zoonotic disease in Chile caused by the dog tapeworm, *Echinococcus granulosus*. In Chile as in many other countries in South America, this disease has been largely neglected with few exceptions. Chile's growing economy and the interest of health authorities has lead to an increase in the number of studies investigating the epidemiology of echinococcosis and the factors related to infections of the main definitive host, the domestic dog and humans. In this study, we determined the prevalence of human and canine echinococcosis as well as the associated risk factors in a rural area of the Limarí province in northern Chile. We undertook a household questionnaire survey in rural areas of the five municipalities of the Limarí province in Coquimbo region. For each household serum of an adult family member and fecal samples from a dog were taken. Results of our study indicate that infection occurs in 2.6% of humans and 28% of dogs and is primarily due to feeding of dogs with contaminated offal and high dog-human contact. As a result of this study, the Chilean Ministry of Health instigated a control program aimed to control the infection in dogs and avoid new infections to humans.

## Introduction

Hydatidosis or cystic echinococcosis (CE) is a chronic zoonotic parasitic disease of almost worldwide distribution caused by the cestode parasite *Echinococcus granulosus*. Cystic echinococcosis accounts for 95% of the estimated 2–3 million cases of human echinococcal infections worldwide and represents a major public health problem in many parts of the world [1]. It is listed by the World Health Organization as a neglected zoonotic disease since this disease mainly affects poor and marginalized populations in low-resource settings (www.who.int/neglected_diseases/zoonoses).

The life cycle of this helminth includes carnivores, mostly dogs, as definitive hosts and herbivores such as sheep and goats as intermediate hosts. Humans become infected after accidental ingestion of eggs excreted with carnivore feces. Cystic echinococcosis in humans and animals is characterized by the development of metacestode larval stages in the liver and other organs. Known key factors of persistence, emergence or re-emergence of hydatid disease in a given human population are among others (i) the presence of large numbers of dogs harbouring *E. granulosus* worms, (ii) access of dogs to infected offal (iii) inadequate facilities for slaughter and destruction of infected viscera, (iv) slaughtering of livestock in homesteads [2], [3]. The practice of feeding dogs with infected offal is by far one of the most important factors for the persistence of this disease [4]. The age of dogs is another relevant factor, since young dogs eliminate higher numbers of echinococcal eggs in their feces than older dogs [5]–[7]. In addition, restrictions regarding dog ownership are of epidemiological influence, since street dogs have an increased risk of acquiring *E. granulosus* infection through uncontrolled access to infected carcasses [7]. Other factors include the lack of regular deworming of dogs and the absence of knowledge pertaining to infection and disease [5]–[7].

*Echinococcus granulosus* is hyperendemic in the southern parts of South America, e.g. Argentina, Chile, Peru, and Uruguay, where it has an important economic and public health impact [8]. Sheep are the main intermediate host for the G1 genotype of *E. granulosus*, which has a worldwide distribution including South America [9]; to the best of our knowledge no studies of the genetic strain circulating in Chile has yet been published. The epidemiological situation in South America is complex and not fully understood and comprehensive epidemiological data is lacking. In Chile, echinococcosis mostly affects humans and their livestock in rural and poorly developed areas. According to national surveillance data, the surgical incidence has remained stable at around 2 cases per 100,000 inhabitants since the early 1990s [10], [11].

Although in Chile hydatid disease is a relevant public health problem, data regarding local distribution and risk factors is limited. One study carried out in the Coquimbo region which assessed risk factors for the presence of *E. granulosus* eggs in dog feces revealed that juvenile dogs from households performing home slaughter, which had not been de-wormed in the two previous months, were at highest risk of contracting echinococcosis [12]. To the best of our knowledge, epidemiological studies of *E. granulosus* infection in both humans and dogs at a household level, which are essential to implement control programs, have not been carried out in Chile and are rarely reported elsewhere [eg. 13], [14], [15]. Our study aimed to determine the prevalence of human echinococcosis as well as the associated risk factors including canine echinococcosis in rural areas of the Limarí province of the Coquimbo region in northern Chile.

## Materials and Methods

### Study Design

**Figure 1:**
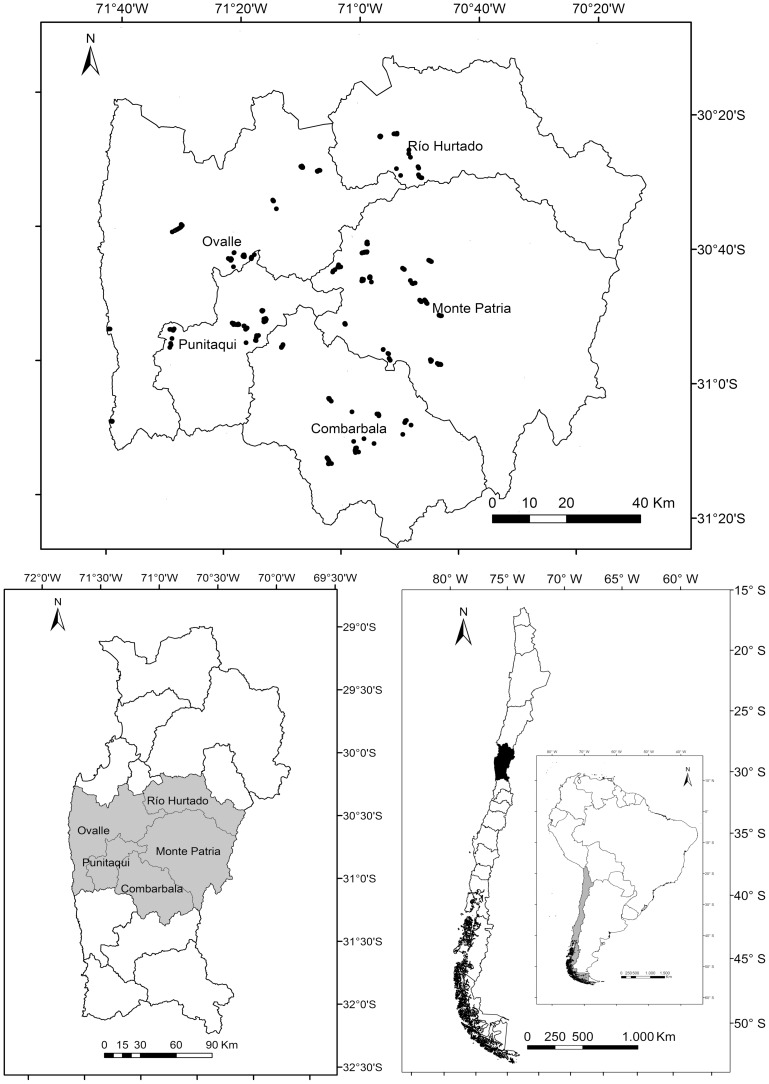
Study site in the Coquimbo region of Chile. Down right: Coquimbo region of Chile (grey); Left: Limarí province within the Coquimbo region with its five municipalities (grey), where the household-based study was performed. Up: Map of the Limarí province. In black dots are interviewed households in the five municipalities across the study area.

A cross-sectional study was conducted from August to November 2009 within the Limarí province of the Coquimbo region in northern Chile (Figure 1). Stratified sampling depended on the number of rural villages in each of the five municipalities. To estimate sample sizes, we used an echinococcosis prevalence of 3% in humans [16] and 28% in dogs [17]. For target values of 90% for confidence intervals and ±2.5% and ±4% for errors of human and dog populations, respectively, a sample size of 480 human samples was estimated using Epi Info 6.0 (wwwn.cdc.gov/epiinfo/). This number was approximated to 500 samples, of which 84, 67, 46, 217, and 86 were assigned to the municipalities of Ovalle, Punitaqui, Rio Hurtado, Monte Patria, and Combarbalá, respectively, according to the proportion of villages of each municipality and the overall number of villages in the province. In each village/settlement, ten households were randomly selected to be visited. During field visits epidemiological data was collected by a standardized questionnaire and blood samples were obtained from one adult per household (keeping an even sex ratio of the total samples). Furthermore, a fecal sample was collected from one dog per household in randomly selected households of two municipalities.

### Ethical Approval

The study was approved by the Institutional Review Board (IRB) of the Ministry of Health at the Coquimbo region. Information regarding the study was initially communicated to potential participants prior to their signing an informed consent.

### Data Collection

The study included a questionnaire survey to determine the potential risk factors for transmission of *E. granulosus* in humans and dogs [eg. 5], [12]. The questionnaire included basic demographical data of the dog owner and his household, data on education and occupation, living standards including waste management and water supply as well as slaughtering practices and knowledge of the disease (using graphic material). Furthermore, the questionnaire covered data about the sampled dogs, dog-keeping practices, such as contact with dogs (i.e. *high*: grooming, petting, sleeping with dogs, dogs allowed to enter into the house; *low*: dogs sleeping outside the household with close contact on few occasions) and other factors that could influence contact between humans and the parasite. Complex questions were asked as open format questions to reduce bias. The questionnaire took between 30–40 minutes to complete.

### Sampling and Laboratory Analysis

After each interview, a blood sample was taken from each participant by peripheral venipuncture. Specimens were centrifuged on the same day using a portable centrifuge (Mobilspine, Vulcon Technologies, Richmond, USA), serum was separated and kept at −20°C until further analysis. Additionally, a fresh fecal sample from one dog within each household was collected either rectally or from the ground as previously described [14]. A small amount (approx. 1 gram) of feces was placed in 5% phosphate-buffered saline formalin and thoroughly mixed. The supernatant was transferred into Eppendorf tubes and maintained at 4°C until further analysis [5].

Human serum samples were all screened by a commercial enzyme-linked immunosorbent assay (ELISA) detecting IgG antibodies against *E. granulosus* antigens (*Echinococcus* IgG ELISA classic, Serion Immundiagnostica, Würzburg, Germany). Positive or indeterminate samples were retested at the Institute of Medical Microbiology, Immunology and Parasitology, Bonn, Germany, by additional serological techniques such as indirect hemagglutination assay (IHA) and immunofluorescence assay (IFA), which have been described elsewhere [18]. Samples with positive or borderline results in either in-house assay were confirmed by Western Blot (WB) (*Echinococcus* Western Blot IgG, LDBIO Diagnostics, Lyon, France). Seropositivity was defined by means of a WB positive result. To diagnose echinococcal infections in dogs, an *Echinococcus* genus-specific coproantigen ELISA was used (Cestode Zoonoses Research Group, University of Salford), as previously described [19], [20].

### Data Analysis

Data was analyzed using Stata 10.0 (Stata Corporation, Texas, USA). To compare frequencies of confirmed seropositive human samples between municipalities, a Fisher exact test was used. Additionally, risk factors associated with seropositivity to *E. granulosus* in people inhabiting rural areas of the Limarí province was assessed by two tailed Fisher or Chi-square tests. To determine risk factors for canine echinococcosis, fixed effect logistic regression analyses were performed, using adjustment by sample numbers in each municipality. For risk analysis, univariate logistic regression was carried out to select variables with p values≤0.250, which were included in further multivariate models.

## Results

### Questionnaire Survey

A total of 393 households were visited (Figure 1). An overall of 3.5 persons and 2.2 dogs per household were included, with a ratio of 1.7 human per each dog per household. Livestock herding was the occupation reported by 23% of interviewees. Only 64% of interviewees reported to have finished their primary education and 52% of them said to have potable water. In 3% of all households, at least one member reported to suffer from hydatid disease. High contact rates with dogs and regular contact with dog feces were reported by 41% and 43% of the participants, respectively. The majority of individuals (75%) stated that they had never dewormed their dogs with antiparasitic drugs. Home slaughter or purchase of undisemboweled animals for consumption was reported in 63% of households, of which 75% had noticed the presence of fluid-filled structures compatible with hydatid cysts. Furthermore, 61% reported the presence of household dogs during slaughter or disembowelment of livestock, 50% reported feeding of dogs with viscera, and 34% feeding of dogs with hydatid cysts. Regarding the knowledge of zoonotic diseases, 55% of participants quoted that they knew of diseases transmitted from animals to humans, however only 17% had heard about human hydatidosis (Table 1).

**Table 1:** Demographic features and behavioral and educational characteristics regarding variables that could be factors for echinococcal infection in the Limarí province, Chile.

Variables	Overall	90% CI
Persons per household	3.5	3.3–3.7
Dogs per household	2.2	2.1–2.3
Human per dog at household	1.7	1.6–1.8
Livestock herding	23%	20%–27%
Primary education	64%	60%–67%
Potable water	52%	48%–57%
Hydatidosis at household	3%	2%–5%
High rate of dog contacts	41%	37%–45%
Collection of dog feces	43%	39%–47%
De-worming of dogs	25%	22%–29%
Home slaughter	63%	59%–67%
Cysts seen in carcasses	75%	71%–78%
Dogs present at slaughter	61%	57%–65%
Feeding dogs with viscera	50%	46%–54%
Feeding dogs with cysts	34%	31%–38%
Zoonotic diseases known	55%	51%–59%
Hydatidosis known	17%	14%–21%

Overall and 90% Confidence intervals are given for each variable.

### Human Seroprevalence and Risk Factors to *E. granulosus*

The initial ELISA screening revealed positive results in 47 out of 403 serum samples, resulting in a seroprevalence of 12% (90% CI 9–14%). When the 47 initially positive samples were retested by IHA, IFA, and WB 10 samples were confirmed positive by this multi-step analysis, resulting in a seroprevalence of 2.6% (90% CI 1.6–4.4%). Prevalence in different municipalities ranged from 0% to 6.8%, but remained without significant differences (Table 2). Statistical analyses revealed that a higher seropositivity was found in people from households reporting high dog contact, those that allowed dogs to defecate in orchards, and those that did not regularly collect their dog feces (Table 3). Due to the small number of positives it was not possible to run a multivariable analysis. A further study with a larger sample size is recommended.

**Table 2:** Seroprevalence of antibodies against *E. granulosus* in different municipalities of the Limarí province, Chile.

Municipality	Total	Positives	Prevalence	90% CI
Combarbalá	60	1	1.7	0.6–6.3
Monte Patria	148	4	2.7	1.3–6.0
Ovalle	77	1	1.3	0.5–5.9
Punitaqui	59	4	6.8	3.3–14.6
Río Hurtado	40	0	0.0	0.0–7.0

ELISA test followed by confirmatory tests.

**Table 3:** Analysis of factors associated with seropositivity to *E. granulosus* in inhabitants of the Limarí province.

Factor	Pos. result	Neg. result	%	p
Sex				
Male	5	125	3.8	
Female	5	249	2.0	0.45
Age				
18–40 years	4	118	3.3	
>40 years	6	256	2.3	0.73
Contact with dogs				
High rate	10	274	3.5	
Low rate	0	97	0.0	**0.07**
Occupation				
Workers	3	121	2.4	
Housewife or retired	6	237	2.5	1.0
Education				
Primary	4	185	2.1	
Secondary	4	165	2.4	1.0
Knowledge of hydatidosis				
No	10	311	3.2	
Yes	0	63	0.0	0.38
Collection of dog feces				
No	9	205	4.2	
Yes	1	168	0.6	**0.04**
Dogs enter domestic area				
No	6	190	3.1	
Yes	4	184	2.1	0.75
Dogs sleep in domestic area				
No	10	346	2.8	
Yes	0	28	0.0	1.0
Dog defecates in backyard				
No	4	307	1.3	
Yes	6	67	8.2	**<0.01**
Regular veterinary care				
No	8	293	2.7	
Yes	2	77	2.6	0.73
Deworming of dogs				
No	7	283	2.4	
Yes	3	91	3.2	0.71
Home slaughter				
No	4	129	3.0	
Yes	6	245	2.4	0.74
Feeding dogs with viscera				
No	5	153	3.2	
Yes	4	220	1.8	0.50
Cysts seen in carcasses				
No	4	150	2.6	
Yes	5	219	2.2	1.0
Public water supply				
No	2	156	1.3	
Yes	8	198	3.9	0.20
Waste disposal				
Within private property	6	185	3.1	
Public collection	3	183	1.6	0.50
Number of dogs in household				
1	4	141	2.8	
>1	6	232	2.5	1.0

Analyses were carried out with Fisher exact test. p<0.1 was considered significant.

### Dog Coproprevalence and Risk Factors to *E. granulosus*

A total of 93 canine fecal samples from the Combarbalá and Monte Patria municipalities were collected and analysed. Of those, 26 were positive resulting in an overall prevalence of 28% (90% CI 21–36%). The prevalence in the municipalities of Combarbalá and Monte Patria was 29% (15/52) and 27% (11/41), respectively.

Variables (n = 15) from the epidemiological questionnaire were tested for the risk of the presence of *E. granulosus* antigen in canine samples in the respective household using a univariable logistic regression analysis (see Table 4). Four variables were selected for further multivariable logistic regression model: ‘*de-worming of dogs*’, ‘*regular veterinary care*’, ‘*home slaughter*’, and *‘feeding dogs with viscera*’ (selected variables in bold in Table 4). Final conditional regression analysis revealed that ‘dogs from households reporting home slaughter of livestock were 3.35 (90% CI 1.16–6.85) times more likely to shed *E. granulosus* antigen than those from households without home slaughter. Furthermore, dogs from households reporting de-worming of pets had a 2.82 (90% CI 1.33–8.43) times higher risk of carrying *E. granulosus* than dogs from households that did not de-worm their dogs.

**Table 4:** Univariable logistic regression analysis of factors associated with *E. granulosus* infection of dogs in households within the Limarí province, Chile (n = 93).

Risk factor	Infected	Uninfected	%	OR	p
Municipality					
Combarbalá	15	37	29	1.00	
Monte Patria	11	30	27	0.90	0.98
Dog's sex					
Male	21	58	27	1.00	
Female	5	9	36	1.53	0.71
Dog's age (months)					
0–12	3	10	23	1.00	
13–24	1	10	9	0.33	0.71
>24	22	47	32	1.56	0.53
Breed					
No	25	59	30	1.00	
Yes	1	8	11	0.30	0.44
Deworming					
No	17	58	23	1.00	
Yes	9	9	50	3.41	**0.04**
Own property					
No	6	13	32	1.00	
Yes	20	53	27	0.82	0.94
Owner's occupation					
Rising livestock, farmer	7	12	37	1.00	
Workers, office, unemployed	1	6	14	0.30	0.38
Housewife, retired	16	48	25	0.57	0.39
Education					
Primary	14	35	29	1.00	
Secondary	8	25	24	0.80	0.85
Graduate	4	6	40	1.67	0.48
Owner knows hydatidosis					
No	18	55	25	1.00	
Yes	8	12	40	2.04	0.28
Regular veterinary care					
No	24	58	29	1.00	
Yes	2	7	22	0.69	**0.24**
Home slaughter					
No	6	31	16	1.00	
Yes	20	36	36	2.87	**0.04**
Cysts seen in carcasses					
No	10	26	18	1.00	
Yes	15	41	18	0.95	0.92
Dogs present at slaughter					
No	12	37	16	1.00	
Yes	14	30	22	1.44	0.58
Feeding dogs with viscera					
Yes	19	25	21	4.56	**<0.01**
Waste disposal					
Within private property	18	41	23	1.00	
Public collection	8	23	14	0.79	0.82

In bold are variables that were retained for the multivariable logistic regression analysis.

## Discussion

Our study was designed to obtain an accurate picture of human and dog echinococcosis in rural areas of the Limarí province in the Coquimbo region through the stratification and random selection of villages and households in each municipality. Based on the seroprevalence of 2.6% it may be assumed that about 1560 individuals in this province would have been in contact with the parasite and could suffer from hydatidosis. This value is several orders of magnitude higher than that officially reported [21] and should be considered when planning control programs.

Our findings confirm that monitoring the seroprevalance by means of an ELISA which applies native, cyst fluid antigens of *E. granulosus* might easily over estimate human exposure to *E. granulosus* in areas where contact or infection with other helminths is possible. Using a one-step approach with an ELISA of low specificity, as done in many epidemiological studies, the seroprevalence rate would have increased to 12% in our population. Through the implementation of a combination of confirmatory techniques, we attempted to eliminate false positive results caused by cross-reactivity with other parasites [4], as suggested by the WHO [22].

Serological studies have been used to assess prevalence and risk factors of cystic echinococcosis in humans worldwide [eg. 14], [23], [24]. Still, this approach does not detect all cases of CE in a population due to its lower sensitivity compared to field studies using imaging techniques such as portable ultrasound [14], [25], [26]. Therefore, we were not able to calculate the number of false negative and positive individuals in this specific area. Future research on CE in the Coquimbo region should include those tests.

Using coproantigen testing we detected a 28% prevalence of canine infection in two municipalities of the Limarí province. A previous study reported a coproprevalence of 7.2% in dogs originating from both rural and urban settings from the Elqui province in the northern part of Coquimbo region [12]. These dogs could be at a lower risk of infection than those in this study and/or are regularly de-wormed by their owners. Nevertheless, the prevalence reported in this study is lower than that found in other rural areas in Latin America, for example in the central Andes of Perú (51%) [25] and in Río Negro in Argentina (42%) [27].

Two risk factors for *E. granulosus* infection of dogs were identified. The first, home slaughter, has been previously reported in Chile [12] and elsewhere [28]. The second factor was previous de-worming of dogs in the respective households. The significance of this finding is unclear; one explanation could be that households which deworm dogs actually have higher risk of parasitic infections and that treatment was irregular and inadequate, e.g. by using pyrantel, which does not eliminate *E.granulosus* tapeworms. Further studies are required to verify this finding such as cross-sectional studies with a larger sample size or longitudinal studies using different de-worming strategies [eg. 29].

In most areas of our study, community level risk factors for the persistence of the parasite within the environment were present such as home slaughter (60%), feeding dogs with cysts (21–45%), and high rates of echinococcosis in dogs (28%). In an endemic area such as the Limarí province, where the main factors for seropositivity are those linked to contact with dogs, it is extremely important for intervention activities to prioritize on the interruption of the chain of infection from dog to human. Thus, strategies should be based on education to promote proper hygienic measures such the management of waste, waste handling, washing hands, the use of plastic gloves when cleaning homesteads, reduction of dog grooming to prevent contact in a highly polluted environment and regular deworming of dogs, repeated at least every 45 days to be effective against *E. granulosus* [1]. This latter strategy is rarely adapted mainly due to economic constraints. Therefore an important intervention would be to increase the frequency of antiparasitic treatment of dogs by governmental sponsored programs, according to international criteria. Still, only a comprehensive program that includes various measures including education and animal management would allow disruption of the cycle of the parasite [30]. Currently, the Chilean Ministry of Health has taken these recommendations into account and instigated a control program with a focus on public education and de-worming and sterilization of dog populations. This latter measure could reduce the contamination of the environment particularly by young dog populations that are known from previous studies to shed large numbers of echinococcal eggs [7].

The results of our questionnaire survey showed that the crucial factors for the maintenance of the life cycle of *E. granulosus* were widely present throughout the rural areas of the Limarí province. In the Coquimbo region, hydatid disease is endemic mainly by the existence of large numbers of goats and sheep maintained in rural areas [31]. Due to poverty and poor animal health management, these areas provide ideal conditions for the maintenance of the life cycle of this parasite. A general limitation of our analysis of risk factors was that the questionnaire did not clearly identify current and past practices, a fact which might confound our results and interpretations.

Cystic echinococcosis is a relevant public health and an economic problem worldwide [15], [32]–[34] as well as in many areas of South America [8] including Chile. [21]. However, due to the lack of solid epidemiological data, difficulties in diagnosis and the chronic nature of infection and the complicated treatment required, it often has low priority and is therefore part of the group of neglected diseases [22]. The epidemiology of human cystic echinococcosis is complex and depends on the presence of the parasite in zoonotic cycles. Prevention and control of infection therefore requires careful mapping of regional epidemiological data and risk factors to tailor intervention strategies to specific situations. Accordingly, this study provides epidemiological data on prevalence and risk factors to both human and canine echinococcal infection that were determined at the household level.

## Supporting Information

Checklist S1 STROBE checklist.
(DOC)
